# CAREGIVER NEGLECT IN OLDER ADULTS WITH COGNITIVE IMPAIRMENT: THEMES FROM EXAMINATION OF CASES

**DOI:** 10.1093/geroni/igae098.2921

**Published:** 2024-12-31

**Authors:** Amy Shaw, Alyssa Elman, Tony Rosen

**Affiliations:** Weill Cornell Medicine, New York, New York, United States; Weill Cornell Medicine, New York, New York, United States; Weill Cornell Medicine, New York, New York, United States

## Abstract

Cognitively impaired older adults are at higher risk for caregiver neglect than other older adults. Characteristics of these neglected older adults and the common themes and surrounding circumstances have not been well described. We used case records from a hospital-based elder mistreatment response team to describe themes in caregiver neglect among older adults with cognitive impairment. We quantitatively and qualitatively examined records from 146 older adults with cognitive impairment for whom the response team found moderate or high suspicion for caregiver neglect. The median age was 82 (IQR 75–90) years; 53.4% were white, 19.2% were Black/African American, and 8.2% were Asian/Asian American; 21.9% had Hispanic ethnicity. Most patients (113, 77.4%) were women. Suspicion of at least one other form of elder mistreatment (verbal/emotional, physical, financial, or sexual abuse) was present for 48.67% of patients. The neglecting caregiver was most often the patient’s child (42.5%), partner/spouse (19.9%), or aide (15.1%). We identified seven key themes about neglect: 1) delays in seeking medical care, 2) inadequate supervision, 3) attempts to honor patient preferences, 4) conflict between caregivers, 5) caregiver impairment (various types), 6) interference with medical or social care, and 7) “mystery friend” or relative moving into patient’s home. Our work suggests that neglected older adults with cognitive impairment are commonly also victims of other forms of elder mistreatment. Themes in elder neglect may be identified that offer insight into the phenomenon. Multiple types of intervention may be needed to address the wide range of situations that may lead to neglect.

